# A behaviour change intervention to reduce home exposure to second hand smoke during pregnancy in India and Bangladesh: a theory and evidence-based approach to development

**DOI:** 10.1186/s40814-021-00811-5

**Published:** 2021-03-19

**Authors:** Veena A. Satyanarayana, Cath Jackson, Kamran Siddiqi, Prabha S. Chandra, Rumana Huque, Mukesh Dherani, Shammi Nasreen, Pratima Murthy, Atif Rahman

**Affiliations:** 1 Department of Clinical Psychology, National Institute of Mental Health And Neuro Sciences (NIMHANS), Bangalore, 560029 India; 2 Valid Research Limited, Sandown House, Sandbeck Way, Wetherby, West Yorkshire LS22 7DN UK; 3 Department of Health Sciences, University of York, Seebohm Rowntree Building, Heslington, York, Y010 5DD UK; 4 Department of Psychiatry, National Institute of Mental Health And Neuro Sciences (NIMHANS), Bangalore, 560029 India; 5 Department of Economics, University of Dhaka and ARK Foundation, House No 6, Road NO 109, Gulshan 2, Dhaka, Bangladesh; 6 Institute of Psychology, Health and Society, University of Liverpool, Liverpool, L69 3BX UK; 7 ARK Foundation, House No 6, Road NO 109, Gulshan 2, Dhaka, Bangladesh

**Keywords:** Behaviour change intervention, Secondhand smoke, Smoke exposure at home, Pregnancy, LAMI

## Abstract

**Background:**

Home exposure to secondhand smoke (SHS) is highly prevalent amongst pregnant women in low- and middle-income countries like India and Bangladesh. The literature on the efficacy of behaviour change interventions to reduce home exposure to SHS in pregnancy is scarce.

**Methods:**

We employed a theory and evidence-based approach to develop an intervention using pregnant women as agents of change for their husband’s smoking behaviours at home. A systematic review of SHS behaviour change interventions led us to focus on developing a multicomponent intervention and informed selection of behaviour change techniques (BCTs) for review in a modified Delphi survey. The modified Delphi survey provided expert consensus on the most effective BCTs in reducing home exposure to SHS. Finally, a qualitative interview study provided context and detailed understanding of knowledge, attitudes and practices around SHS. This insight informed the content and delivery of the proposed intervention components.

**Results:**

The final intervention consisted of four components: a report on saliva cotinine levels of the pregnant woman, a picture booklet containing information about SHS and its impact on health as well strategies to negotiate a smoke-free home, a letter from the future baby to their father encouraging him to provide a smoke-free home, and automated voice reminder and motivational messages delivered to husbands on their mobile phone. Intervention delivery was in a single face-to-face session with a research assistant who explained the cotinine report, discussed key strategies for ensuring a smoke-free environment at home and practised with pregnant women how they would share the booklet and letter with their husband and supportive family members.

**Conclusion:**

A theory and evidence-based approach informed the development of a multicomponent behaviour change intervention, described here. The acceptability and feasibility of the intervention which was subsequently tested in a pilot RCT in India and Bangladesh will be published later.

**Supplementary Information:**

The online version contains supplementary material available at 10.1186/s40814-021-00811-5.

## Key messages regarding feasibility

- We developed a theory and evidence-based behaviour change intervention to reduce home exposure to secondhand smoke in pregnant women.
- Findings from a systematic review, a modified Delphi survey and qualitative interviews with key informants informed the development of our multicomponent behaviour change intervention.
- The next step is to test the feasibility and acceptability of the intervention in a pilot RCT in India and Bangladesh.

## Background

Over one-third of all women, globally, are exposed to secondhand smoke (SHS) [1–3]. In low- and middle-income (LAMI) countries, most SHS exposure amongst women in the reproductive age group occurs at home, where women spend most of their time [4, 5]. Estimates of home exposure to SHS have ranged from 17.8% in Mexico to 72.3% in Vietnam [6]. A more recent study [7] using the Demographic and Health Survey data (2008 and 2013) from 30 LAMI countries (*N* = 37,427 pregnant women) found that the weighted country-specific prevalence of SHS exposure ranged from 7% (6–9%) in Nigeria to 81% (72–88%) in Armenia. More than 50% of pregnant women reported some (daily, weekly, monthly or less than monthly) SHS exposure in five countries (Jordan, Armenia, Bangladesh, Indonesia and Nepal), and more than 50% of pregnant women reported daily SHS exposure in three countries (Jordan, Armenia and Indonesia). Pregnant women in the Southeast Asian countries had the highest probability of exposure. Those in urban areas had a higher probability for household SHS exposure than pregnant women in rural areas. Exposure to SHS during pregnancy is associated with a range of adverse maternal and infant health outcomes such as pregnancy complications, low birth weight, still birth, small for gestational age infants and sudden infant death syndrome [8–13].

Studies have speculated that women in China, Cambodia and India may often be unable to negotiate a smoke-free home with their husbands possibly due to patriarchy, gender inequity and gendered power interactions [9, 14, 15]. Additional factors include low literacy levels, lack of awareness about the possible dangers of home exposure to SHS and culturally held beliefs about men’s smoking behaviours. A typical example of such beliefs is that smoking helps them unwind after a long day’s work, which prevents negotiation for a smoke-free home [8–10, 16–18]. For example, a study from China demonstrated that despite women holding negative attitudes towards smoking, they either rationalized men’s smoking or chose not to assert their views for fear of causing conflict at home [19]. The World Health Organization (WHO) provides guidelines recommending antenatal care providers to routinely screen pregnant women for tobacco use and home exposure to SHS and suggests strategies for smoking cessation and prevention of home exposure to SHS [20]. Intervention studies on reducing home exposure to SHS have included a range of education and counselling/brief advice strategies delivered by health workers to create awareness, enhance knowledge about its harms, attempt attitudinal change and suggest practical methods of ensuring a smoke-free home [21]. Very few studies have, however, included strategies that allow the woman to negotiate a smoke-free home with significant male family members [22]. Our work aimed to develop a multicomponent intervention that incorporated this strategy (focusing particularly on the pregnant women’s husbands) alongside other established behaviour change techniques (BCTs) [23] to allow a comprehensive approach to reducing exposure to SHS in the home environment during pregnancy.

## Methods

**Fig. 1:**
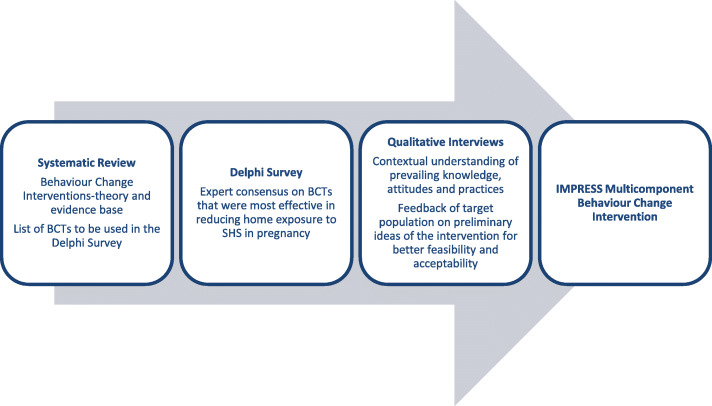
The three approaches that informed the development of IMPRESS multicomponent behaviour change intervention

We adopted a theory and evidence-based approach to intervention development [24, 25]. We conducted a systematic review to obtain a critical understanding of the evidence base, a modified Delphi survey to obtain expert consensus on effective BCTs and qualitative interviews for contextual understanding of knowledge, attitudes and SHS practices. The key findings from each of these three complementary studies informed the development of the IMPRESS (*I*ntervention for *M*others during ***P***regnancy to *R*educe *E*xposure to *S*econd hand *S*moke) intervention at a workshop held in Dhaka, Bangladesh (see Fig. 1).

### Systematic review (detailed methods described elsewhere [22])

The systematic review (a) reported the behaviour change interventions for reduction in home exposure to SHS in pregnant women and (b) critically appraised intervention reporting, as well as generalisability, feasibility and scalability of these interventions. It identified six studies for inclusion. These studies evaluated interventions targeting pregnant women, delivered in antenatal clinics, at home, by telephone or a mix of these. They focused on education about SHS and/or developing skills in women to avoid SHS exposure or negotiate with a family member, usually the husband. Five interventions were underpinned by a behaviour change framework, for example, the Transtheoretical Model of Change [26] and the Health Belief Model [27].

We present below the contribution of (a) to our intervention development. An important observation was that the evidence was insufficient to provide guidance on the essential components of the IMPRESS intervention indicating the need for a modified Delphi Survey to obtain expert consensus on effective BCTs in reducing home exposure to SHS.

Regarding (b), reporting of the intervention studies did not meet the Workgroup for Intervention Development and Evaluation Research (WIDER) guidelines for reporting of behaviour change interventions [28] and no studies met all generalizability, feasibility and scalability criteria. Whilst these findings were not relevant to the development of the IMPRESS intervention, they highlighted the importance of detailed reporting of the development process, its theoretical underpinning and subsequent evaluation.

### Modified Delphi survey

This was conducted to build consensus amongst international experts and identify the most effective BCTs to reduce home exposure to SHS in pregnant women. Our approach differed from the original Delphi method in that independent opinion was sought via email rather than face-to-face consultation with a group of experts, and an evidence-based list of BCTs was generated by the investigators and emailed to the experts [25]. This is a time and cost-efficient method of achieving consensus amongst international experts [29, 30].

#### Sample

The sample comprised of experts who were lead authors of peer-reviewed international publications in the areas of smoking cessation, SHS and behaviour change interventions. We attempted to have global representation. Through a process of discussion and elimination, we identified a final group of 30 experts who were contacted via email requesting their participation in the survey. We had experts participate from both LAMI (Bangladesh, India, Pakistan, China) and high-income countries (USA, UK, Canada, Australia).

#### Procedure

A seminal publication on BCTs [23], our systematic review [22] and a recent paper on BCTs in waterpipe smoking [31] were used to generate a list of BCTs that were most relevant to reduction of home exposure to SHS. Initially, 32 BCTs were short listed by VS of which 21 BCTs were rated by two members of the research team (VS, KS *Kappa* = 0.92) as most relevant to reduction of SHS at home during pregnancy. The BCTs that were eliminated at this stage focused primarily on smoking cessation rather than reduction of home smoking alone. The 21 BCTs included enhancing knowledge and awareness, making an appraisal of risks and benefits, and using specific strategies such as prompts, problem solving, negotiation etc. (see Additional File 1). Three rounds of Delphi were chosen a priori to reach an acceptable consensus.

In the first round of the Delphi, 30 experts were requested to rank in the order of preference the most effective BCTs that in their opinion were likely to reduce home exposure to SHS. To aid their judgement of importance, they were requested to consider acceptability, deliverability and efficacy of each BCT. Their responses were anonymous. As background information, experts were informed that our proposed multicomponent intervention was likely to include two methods of intervention delivery: communicating with the pregnant woman (non-smoker and the primary participant at the health clinic) and with her husband (smoker and the secondary participant) possibly through digital/mobile phone technology.

In round 2, experts who participated in round 1 were given feedback about the opinion of the whole group (e.g. average rank assigned for each BCT) and asked to re-evaluate their original ranking in view of this information. This was repeated in the final round 3. On average, two reminders were sent to the experts requesting them to turn in their ratings of BCTs.

### Qualitative interviews (detailed methods described elsewhere [18])

Key informant interviews (*N* = 64) were carried out with pregnant women, husbands who smoked at home, husbands who did not smoke at home, and family members (parents, in-laws etc.) in India and Bangladesh to understand contextual determinants of home exposure to SHS, knowledge attitudes and SHS practices. The focus of the interviews was the smoking behaviour of pregnant women’s husbands although details of other family members’ smoking in the home also featured in participants’ accounts. Interviews were conducted in Comilla (rural Bangladesh) and in Bangalore (urban India) to ensure relevance to both rural and urban settings.

## Results

The detailed findings of the systematic review and qualitative interviews are published elsewhere [18, 22]. How these two studies informed the IMPRESS intervention development is described below and in Table 1.

**Table 1:** Multicomponent behaviour change intervention informed by the systematic review, modified Delphi survey and qualitative interviews

Informed by the systematic review	Selected BCTs from modified Delphi survey	Context and detail from qualitative interviews	Intervention content and delivery (intervention component)
Decision to develop a multicomponent behaviour change intervention and 14 BCTs taken forward for inclusion in the modified Delphi survey	Measure cotinine (marker for SHS exposure) in non-smokers and give feedback	Pregnant women, husbands and family members have poor understanding of the health risks of SHS to the health of the pregnant women and their future child. Pregnant women and family members think educating their husbands about the risks of his smoking to his future child may change his behaviour. Husbands agree this would motivate them. The source of this education is seen as important with university employees or health professionals seen as more credible (and influential) than the pregnant woman.	Personalized feedback on the impact of SHS on the pregnant woman (and therefore her future child) is presented in an ‘official report’ (cotinine report).
	Information about health consequences of SHS and of smoking restrictions at home		Story provides information on the health consequences of SHS to the entire family and the benefits of smoking restrictions in the home (picture booklet). Feedback on the impact of the husband’s smoking in the home on his future child is directly targeted at the husband (letter from the future child).
	Information about social and environmental consequences	Pregnant women lose confidence in asking their husbands to smoke outside. Some are frightened of his reaction.	The story shows the husband being receptive to discuss this with his wife (picture booklet). Husbands are encouraged to discuss with their wives the steps they could take to make their home smoke free (voice messages).
	Salience of consequences	Husbands do not acknowledge the impact of their smoking inside.	Emotive language directed at the husband is used (letter from the future child) and the story included pictures showing the impact on his entire family (picture booklet).
	Identify reasons/motives for wanting and not wanting to stop smoking inside homes	Pregnant women dislike the smell of smoke, feel nauseous and struggle to breathe. They want a smoke-free home for their own and children’s health (also a motive for some husbands). Most husbands enjoy smoking in their home surrounded by family. They do not want to be seen smoking outside, dislike the cold and insects and fear fines/for their safety. Clear consensus amongst pregnant women, husbands and family members that the husband’s priority is his children including the future child.	Story shows the pregnant woman and her husband sitting together to discuss the husband’s smoking and reasons why he should stop smoking in the home. Reference is made to the harms to children and future child from their father’s smoking indoors. Positive images of a smoke-free home, highlighting multiple benefits are depicted (picture booklet). Feedback about the impact of the husband’s smoking in the home on his future child is directly targeted at the husband (letter from the future child).
	Facilitate barrier identification and problem solving	Pregnant women repeatedly ask their husbands to smoke away from them and their children, or to smoke outside, with little success. They feel frustrated and often decide to give up. Husbands agree they usually ignore these requests.	Story shows the pregnant woman and her husband sitting down together to discuss the barriers to him smoking outside. There is an action plan for them to complete together (picture booklet). Husbands are reminded to take steps to make their home smoke free (voice messages).
	Prompt practice	Pregnant women report feeling unsupported by family members in challenging husbands’ smoking behaviours. They lose confidence to negotiate with their husbands and some are frightened of his reaction. Conversely, most husbands do not believe it is hard for their wives to request them to smoke outside. Pregnant women think that if other family members, especially elders, ask the husbands to smoke outside, this may be successful. Requests from their children were also seen as potentially influential.	Story shows the pregnant woman asking for support from her family members to ask her husband to smoke outside. Women are instructed to enlist support from their own family members to negotiate with their husband (picture booklet).

### Systematic review

The review concluded that multicomponent behaviour change interventions and their constituent education and skills-based strategies (BCTs) appeared effective in reducing SHS exposure during pregnancy. This informed our decision to use a multicomponent behaviour change intervention using BCTs. However, a small evidence base and weak study methodology (self-reported exposure, lack of objective outcome assessment, short follow-up, absence of control group) prevented firm conclusions about the specific BCTs to employ. Instead, 14 BCTS employed in the six intervention studies were included in the list of 21 BCTs presented to experts in round 1 of the Delphi survey (see Additional File 1).

### Modified Delphi survey

In round 1 of the Delphi, of the 30 experts contacted, 17 experts (57% response rate) turned in their responses via email. These 17 experts were contacted for round 2, of whom 15 experts turned in their rankings (88% response rate). In the final round, the same 15 experts turned in their rankings (100% response rate). Consensus was assessed using Kendall’s *W* statistics where < 0.5 indicated poor consensus, 0.6–0.8 indicated moderate consensus and > 0.8 was strong consensus. Consensus achieved in each round is summarized in Table 2.

**Table 2:** Kendall’s *W* coefficient across the three rounds of Delphi

	Round 1 (***N*** = 17)	Round 2 (***N*** = 15)	Round 3 (***N*** = 15)
**Kendall’s** ***W***	0.25 (< 0.001)	0.43 (< 0.001)	0.61 (< 0.001)

The seven BCTs (see Table 1) that were most preferred by experts in round 3 were then used to guide the development of the IMPRESS intervention.

### Qualitative interviews

The interview findings were revisited to provide detail for the seven selected BCTS as ingredients of the IMPRESS intervention components (see Table 1). As an example, for the BCT ‘identify reasons/motives for wanting and not wanting to stop smoking inside homes’, pregnant women disliked the smell of smoke, felt nauseous and wanted a smoke-free home for their own health and that of their children/future child. Some husbands wanted to quit smoking in their home to protect their children and future child, although most liked smoking in the comfort of their own home, surrounded by their family. They did not want to be seen by others when smoking outside and mentioned concerns about the cold, insects, personal safety and being fined. The consensus amongst pregnant women, husbands and family members was that the husband’s priority is his children including the future child. This detail was used to develop positive images of a smoke-free home highlighting the cited benefits. In addition, feedback about the impact of the husband’s smoking in the home on his future child directly targeted the husband.

### Development of the intervention

The findings of the three studies described above were discussed at an intervention development workshop in Dhaka, Bangladesh (September 2016), where the research team participated in intensive week-long deliberations. During this workshop, three team members leading one of the three studies presented their key findings to the team. Following the presentations, relevant findings from each of the three studies that informed content and delivery were extracted through discussion and consensus amongst team members was achieved (resulting in Table 1).

A working draft of the content and delivery of our proposed multicomponent IMPRESS intervention was created and reviewed to ensure it could be feasible, scalable, sustainable, gender and culturally relevant and cost-effective. An additional consideration was to ensure that the intervention could be delivered to people with low literacy. This was identified as a limitation in existing SHS interventions [21, 22] and a priority for our target audience.

A team of illustrators, graphic designers and technology partners were later involved to ensure that the content and delivery of health messages were impactful.

### Intervention content

**Fig. 2:**
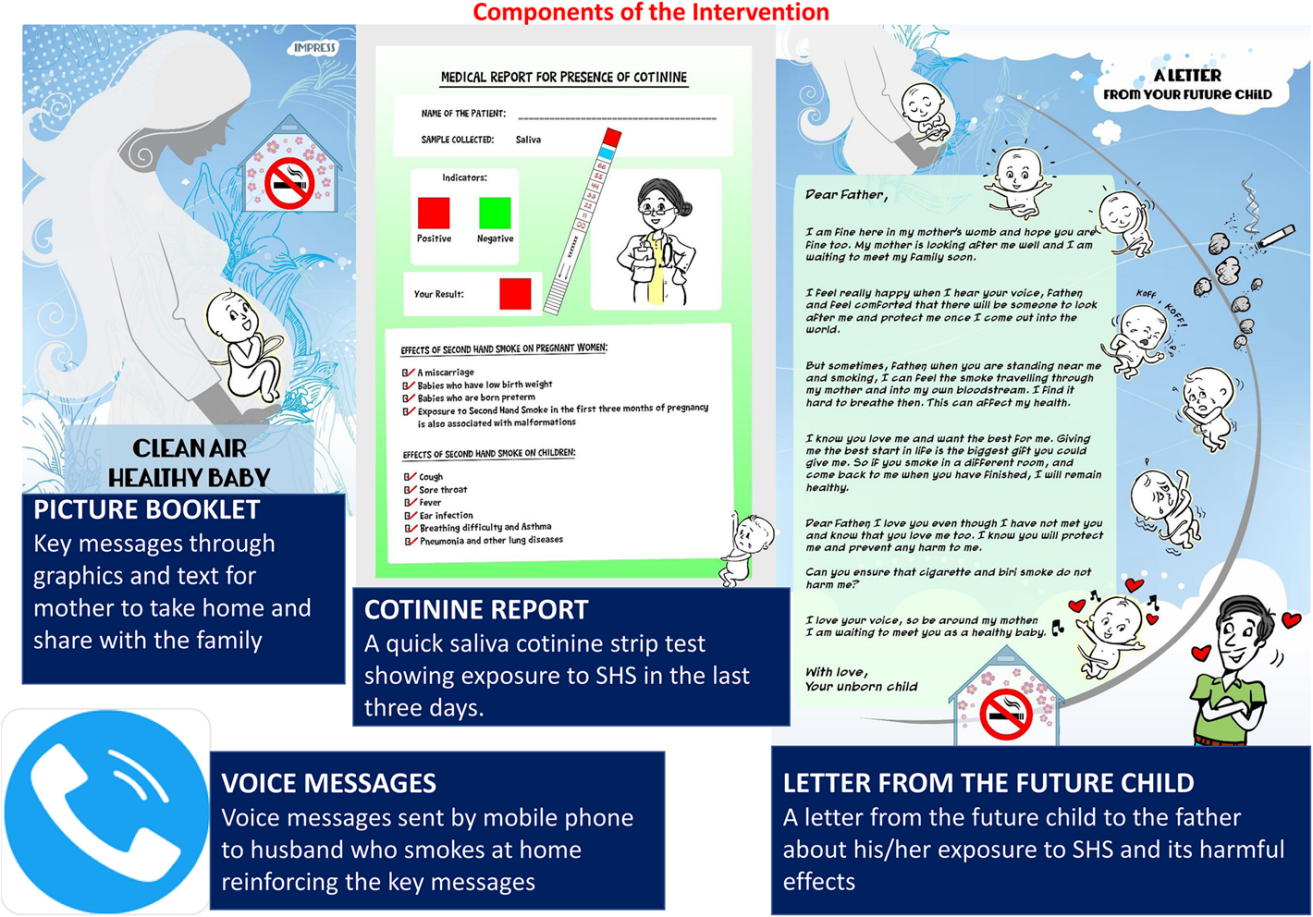
IMPRESS multicomponent behaviour change intervention

The four components of the IMPRESS multicomponent behaviour change intervention are now described (see Fig. 2).

#### Picture booklet

The picture booklet titled “Clean air, healthy baby” consists of a combination of graphics and text description on topics relevant to reduction of SHS at home. These include but are not limited to (i) knowledge about SHS, (ii) benefits of change, (iii) taking practical steps to reduce smoking at home and (iv) getting the help of others, e.g. family members. It includes a page where the pregnant woman and her husband agree to any three commitments, they choose to make towards a smoke-free home. The picture booklet also includes a pocket to store the cotinine feedback report and letter from the future child described below. It was developed in English and translated to Kannada and Bengali for use in the pilot RCT in India and Bangladesh.

#### Cotinine report

NICALERT, a quick saliva cotinine screening test for exposure to SHS, is a standardized and reliable measure. A saliva sample was collected from women in the antenatal clinic using a funnel and collection container provided. The NicAlertTM test device was laid on a dry flat surface with the numbered levels facing up. The saliva sample was applied to the absorbent cotton wick end of the test strip till it was completely saturated (usually 4–5 drops). Results were read after 20 min. A level above 10 ng/ml indicates a positive test. Objective colour-coded feedback about the presence of cotinine through the NICALERT test is provided in the report.

#### Letter from the future child

The letter from the future child is a rich narrative about their exposure to SHS and its harmful effects on the foetus and mother. This letter is addressed to the father (who smokes at home).

#### Voice messages

Four automated voice messages are to be delivered as per a standard schedule (weekly = 2, fortnightly = 1 and monthly = 1) from the study office to the husband of the pregnant woman. The automated voice messages remind him to read the picture booklet if he has not done so already and to take steps to make their home smoke free.

### Intervention delivery

One face-to-face session with the pregnant woman was planned where the interventionist would briefly go through the contents of the picture booklet. This picture booklet (including the cotinine report and letter from the future child) was subsequently given to the pregnant woman to take home, encouraging her to share it with her husband and family members. A week later, voice messages were delivered to the husband as per the above-described schedule.

### Training of interventionists

Two research assistants with a Master’s degree in psychology/humanities delivered the intervention. A half-day training package was developed. It comprised a brief rationale for the proposed intervention, overview of the multicomponent intervention, do’s and don’ts in the conduct of the intervention, and role plays. Some of the skills and competencies imparted during training included finding the right time and setting to negotiate a smoke-free home, not engaging in blaming the husband rather jointly taking steps to promote a smoke-free home in the interest of the entire family. Specifically, communication and negotiation skills were the key focus.

## Discussion

BCTs are theory-informed and evidence-based strategies aimed at enhancing positive health behaviours [23, 32]. They have received widespread popularity and have an evidence base in reducing smoking behaviours [31, 33]. However, there is little research on behaviour change interventions to reduce home exposure to SHS in pregnancy [21, 22]. Consistent with recommendations [24, 25, 32], we employed theory and evidence-based approach to detail the systematic development of our multicomponent behaviour change intervention (IMPRESS) that was informed by a systematic review, modified Delphi survey and qualitative interviews with key informants. Whilst our approach is described as ‘theory and evidence-based’, it uses the philosophy of other approaches, namely, ‘target population centred’, ‘implementation based’ and ‘efficiency based’ [25]. IMPRESS is also gender and culturally relevant. It is designed to empower the pregnant woman to be the main agent of change of her husband’s smoking behaviour whilst recognizing that this is a significant challenge in developing and patriarchal countries [14, 15, 33].

IMPRESS comprised four components. Cotinine levels in the pregnant women’s saliva were measured as an objective indicator of SHS exposure. Feedback via an ‘official’ cotinine report was designed to educate the pregnant woman and her husband on the health risks of his smoking to the women and the future child. The letter from the future child to the father was written to appeal directly to the husband’s motivation to protect his children. The picture booklet was developed to increase awareness about SHS and its harms; it also offered practical strategies to help the woman discuss smoking with her husband and enlist help from supportive family members to negotiate with her husband. It was simple and self-explanatory to cater to the low literacy levels of our sample but also to be visually appealing, to engage the target audience. Finally, automated voice messages were delivered to the husband to encourage him to read the picture booklet and discuss with his wife how he could take steps to make their home smoke free. Voice messages have been under-utilized in SHS interventions although m-health interventions are known to be cost-effective, scalable and sustainable [21]. Voice messages were used as opposed to text messages, due to the low literacy level of our target population. They were also considered to be more feasible than engaging with the men in person.

The IMPRESS intervention package was designed to be brief and easy to deliver by antenatal staff with minimal training to maximize its scalability and sustainability. In line with WHO’s directive, it could potentially be integrated into routine antenatal care for screening and intervention in these countries where the prevalence of SHS is high [20].

In line with the MRC framework [24], the next step was a pilot RCT to assess the acceptability and feasibility of the IMPRESS intervention in India and Bangladesh. This has recently been completed. The results, to be published soon, will inform plans to conduct a multi-country definitive RCT.

Whilst our approach has many strengths as described above, it also has limitations related to the modified Delphi survey. A moderate consensus amongst experts on the most effective BCTs was achieved after three rounds. This may be because the Delphi panel was heavily skewed towards the UK experts. Although a high consensus is desirable, a moderate one is acceptable in this niche area where there is paucity of research on SHS [31].

## Conclusions

A theory and evidence-based approach informed the development of a multicomponent behaviour change intervention informed by a systematic review, modified Delphi method and qualitative interviews. The intervention has subsequently been evaluated in a pilot RCT for its feasibility and acceptability in two LAMI countries, India and Bangladesh, where the prevalence of home exposure to SHS is high.

## Supplementary Information

**Additional file 1:.** Behaviour Change Techniques and examples in relation to SHS

## Data Availability

The datasets used and/or analysed during the current study are available from the corresponding author on reasonable request.

## Abbreviations

SHS: Secondhand smoke
LAMI: Low and middle income
BCTs: Behaviour change techniques
RCT: Randomized controlled trial
WHO: World Health Organization

## References

[1] World Health Organisation. Report of the Global Tobacco Epidemic, 2009: implementing smoke-free environments.Geneva: World Health Organization. 2009;http://www.who.int/tobacco/mpower/2009/en/(accessed 12 Jan 2013).

[2] Öberg M, Jaakkola MS, Woodward A, Peruga A, Prüss-Ustün A (2011). Worldwide burden of disease from exposure to second-hand smoke: a retrospective analysis of data from 192 countries. Lancet..

[3] Ezzati M, Lopez AD, Rodgers A, Vander Hoorn S, Murray CJ (2002). Selected major risk factors and global and regional burden of disease. The Lancet..

[4] Yang G, Fan L, Tan J, Qi G, Zhang Y, Samet JM, Taylor CE, Becker K, Xu J (1999). Smoking in China. JAMA.

[5] Yang G, Ma J, Liu N, Zhou L (2005). Smoking and passive smoking in Chinese, 2002. Zhonghualiuxingbingxuezazhi = Zhonghualiuxingbingxuezazhi.

[6] Centers for Disease Control and Prevention. Current tobacco use and secondhand smoke exposure among women of reproductive age—14 countries 2008–2010.MMWR.2012;61:877–82. 23114255

[7] Reece S, Morgan C, Parascandola M, Siddiqi K (2019). Secondhand smoke exposure during pregnancy: a cross-sectional analysis of data from Demographic and Health Survey from 30 low-income and middle-income countries. Tob Control..

[8] Deshmukh J, Motghare D, Zodpey S (1998). Low birth weight and associated maternal factors in an urban area. IndianPediatr..

[9] Gupta P, Subramoney S (2006). Smokeless tobacco use and risk of stillbirth: a cohort study in Mumbai, India. Epidemiology.

[10] Goel P, Radotra A, Singh I, Aggarwal A, Dua D (2004). Effects of passive smoking on outcome in pregnancy. J. Postgrad. Med..

[11] Leonardi-Bee J, Britton J, Venn A (2011). Secondhand smoke and adverse fetal outcomes in nonsmoking pregnant women: a meta-analysis. Pediatrics..

[12] Kwok MK, Schooling CM, Ho LM, Leung SSL, Mak KH, McGhee SM, Lam TH, Leung GM (2008). Early life second-hand smoke exposure and serious infectious morbidity during the first 8 years: evidence from Hong Kong’s “Children of 1997” birth cohort. Tob Control..

[13] Yang L, Tong EK, Mao Z, Hu T (2010). Exposure to secondhand smoke and associated factors among non-smoking pregnant women with smoking husbands in Sichuan province, China. Acta Obstet Gynecol Scand.

[14] Lee AH (2008). A pilot intervention for pregnant women in Sichuan, China on passive smoking. Patient Educ Couns..

[15] Zheng P, Berg C, Kegler M, Fu W, Wang J, Zhou X, Liu D, Fu H (2014). Smoke-free homes and home exposure to secondhand smoke in Shanghai, China. Int J Environ Res Public Health..

[16] Venners SA, Wang X, Chen C, Wang L, Chen D, Guang W, Huang A, Ryan L, O’Connor J, Lasley B, Overstreet J, Wilcox A, Xu X (2004). Paternal smoking and pregnancy loss: a prospective study using a biomarker of pregnancy. Am J Epidemiol..

[17] Huang CM, Wu HL, Huang SH, Chien LY, Guo JL (2013). Transtheoretical model-based passive smoking prevention programme among pregnant women and mothers of young children. Eur J Public Health..

[18] Jackson C, Huque R, Satyanarayana V (2016). “He doesn’t listen to my words at all, so I don’t tell him anything”-a qualitative investigation on exposure to second hand smoke among pregnant women, their husbands and family members from rural Bangladesh and urban India. Int J Environ Res Public Health.

[19] Mao A, Bristow K, Robinson J (2013). Caught in a dilemma: why do non-smoking women in China support the smoking behaviors of men in their families?. Health Educ Res..

[20] World Health Organization. WHO recommendations for the prevention and management of tobacco use and second-hand smoke exposure in pregnancy. World Health Organization; Geneva, Switzerland: 2013; (accessed on 20 July 2016), Available online: http://www.who.int/tobacco/publications/pregnancy/guidelinestobaccosmokeexposure/en. 24649520

[21] Tong TV, Dietz PM, Rolle IV, Kennedy SM, Thomas W, England LJ (2015). Clinical interventions to reduce secondhand smoke exposure among pregnant women: a systematic review. Tob Control.

[22] Dherani M, Zaidi SN, Jackson C (2017). Behaviour change interventions to reduce second-hand smoke (SHS) exposure in pregnant women – a systematic review and Intervention appraisal. BMC Pregnancy Childbirth.

[23] Michie S, Richardson M, Johnston M, Abraham C, Francis J, Hardeman W, Eccles MP, Cane J, Wood CE (2013). The behavior change technique taxonomy of 93 hierarchically clustered techniques: building an international consensus for the reporting of behavior change interventions. Ann BehavMed.

[24] Craig P, Dieppe P, Macintyre S, Michie S, Nazareth I, Petticrew M (2008). Developing and evaluating complex interventions: the new Medical Research Council guidance. BMJ.

[25] O’Cathain A, Croot L, Sworn K, Duncan E, Rousseau N, Turner K, Yardley L, Hoddinott P (2019). Taxonomyof approaches to developing interventions to improve health: a systematic methods overview. Pilot Feasibility Stud..

[26] Hashemzadeh M, Rahimi A, Zare-Farashbandi F, Alavi-Naeini AM, Daei A (2019). Transtheoretical model of health behavioral change: a systematic review. Iran J Nurs Midwifery Res..

[27] Jones CJ, Smith H, Llewellyn C (2014). Evaluating the effectiveness of health belief model interventions in improving adherence: a systematic review. Health Psychology Review..

[28] Albrecht L, Archibald M, Arseneau D, Scott SD (2013). Development of a checklist to assess the quality of reporting of knowledge translation interventions using the Workgroup for Intervention Development and Evaluation Research (WIDER) recommendations. Implement Sci..

[29] Black N, Murphy M, Lamping D, McKee M, Sanderson C, Askham J, Marteau T (1999). Consensus development methods: a review of best practice in creating clinical guidelines. J Health Serv Res Policy..

[30] Fink A, Kosecoff J, Chassin M, Brook RH (1984). Consensus methods: characteristics and guidelines for use. Am J Public Health..

[31] O’Neill N, Dogar O, Jawad M, Keller I, Kanaan M, Siddiqi K (2018). Which behavior change techniques may help waterpipe smokers to quit? An expert consensus using a modified Delphi technique. Nicotine Tob Res..

[32] Michie S, Fixsen D, GrimshawJM EMP (2009). Specifying and reporting complex behaviour change interventions: the need for a scientific method. Implement Sci.

[33] Robertson S, Williams R (2007). Masculinities, men and public health policy. Int J Interdisciplinary Soc Sci.

