# Supplementary material for: A behaviour change intervention to reduce home exposure to second hand smoke during pregnancy in India and Bangladesh: a theory and evidence-based approach to development
Source: Pilot Feasibility Stud. 2021 Mar 19;7:74. doi: 10.1186/s40814-021-00811-5 (PMC7977562; doi:10.1186/s40814-021-00811-5)
Supplement: Supplementary file 1 — Additional file 1:. Behaviour Change Techniques and examples in relation to SHS [file 40814_2021_811_MOESM1_ESM.docx]

| **Sl No** | **Behaviour Change Techniques** | **Examples in relation to SHS** |
| --- | --- | --- |
| 1* | Provide information on the consequences of SHS and the consequences of smoking restrictions at home | Give salient information about the harm caused by SHS and the benefits of smoking restrictions at home; debunk myths (e.g. passive smoking is not harmful), challenge perception that passive smoking is not harmful, give information on health benefits of smoke free homes and smoking restrictions particularly during pregnancy |
| 2* | Information about social and environmental consequences | E.g. Inform about risks to family/children/pregnant women from smoking at home |
| 3 | Identify reasons/motives for wanting and not wanting to stop smoking inside homes | Develop a clear understanding of his or her feelings about common reasons why he smokes at home? Why it is important to stop? and any conflicting motivations and use these to educate |
| 4 | Incompatible beliefs | E.g. Gently challenge and address beliefs that may be incompatible with smoking at home |
| 5* | Measure cotinine (marker for SHS exposure) in non-smokers and give feedback | Measure cotinine (marker for SHS exposure) in pregnant woman and give feedback to their husband and other family members |
| 6* | Salience of consequences | Use powerful images or techniques to emphasise the harmful consequences of SHS exposure to children and pregnant women |
| 7 | Credible source/Prestige Suggestion | E.g. Present a clip (video) by a high status professional explaining the risks of SHS exposure |
| 8* | Pros and cons | Advise the person to list and compare the advantages and disadvantages of smoking at home e.g. social, emotional |
| 9* | Comparative imagining of future outcomes | E.g. Imagine the future if you stop SHS at home and compare to imagining a future where you do not stop SHS at home. Imagine what it would be like to live in a Smoke Free Home |
| 10* | Advice on changing routine | E.g. Suggest to a person to not keep cigarettes at home visible. Removing ashtrays, Go out of the house when need a smoke |
| 11* | Advice on environmental restructuring | Advise on ways of changing the physical environment to minimise exposure to smoke (e.g. Smoke on terrace/outdoors) |
| 12* | Restructuring the social environment | E.g. Advise to not socialise with friends who prefer to smoke at home |
| 13 | Facilitate barrier identification and problem solving | Identify general barriers (e.g. to unwind after a long day’s work) that might make it harder to stay off from smoking at home and develop general ways of addressing these |
| 14 | Behaviour substitution | E.g. Suggest that the person goes for a walk and then smoke if he wants to rather than smoking at home |
| 15* | Set graded tasks | Set small achievable goals where appropriate (e.g. smoke at home but away from mother/baby, smoke in the bathroom, smoke outdoors) |
| 16* | Prompt commitment from the client there and then | Encourage smoker to affirm or reaffirm commitment to smoke outside home. Using a written promise and positive self talk |
| 17 | Action planning | Planning how you will do the behaviour (e.g. When I come under pressure to smoke at home, I will buy time and try to delay the urge, or, I will go outside/to the terrace/to any open space close by to smoke) |
| 18 | Behavioural contract | Sign a contract with the person e.g. specifying that they will not smoke at home from now on |
| 19* | Teach to identify prompts or cues | Help identify cues ( may be different modalities-visual, auditory etc or contextual) that remind a person to smoke |
| 20* | Prompt practice | Pregnant woman can rehearse negotiation with spouse before actually speaking to her spouse about smoke free home. |
| 21* | Use follow up prompts | Spouse may be sent follow up text messages to remind him about ensuring a smoke free home |

*Derived from the systematic review
